# Supplementary material for: High Proportion of Potential Candidates for Immunotherapy in a Chilean Cohort of Gastric Cancer Patients: Results of the FORCE1 Study
Source: Cancers (Basel). 2019 Aug 30;11(9):1275. doi: 10.3390/cancers11091275 (PMC6770659; doi:10.3390/cancers11091275)
Supplement: Supplementary file 1 [file cancers-11-01275-s001.zip › Supplementary Tables and Figures.pdf]

Supplementary Materials

High Proportion of Potential Candidates for Immunotherapy in a Chilean Cohort of Gastric Cancer Patients: Results of the FORCE1 Study

Miguel Cordova-Delgado, Mauricio P. Pinto, Ignacio N. Retamal, Matías Muñoz-Medel, María Loreto Bravo, Mariía F. Fernández, Betzabé Cisternas, Sebastian Mondaca, Cesar Sanchez, Hector Galindo, Bruno Nervi, Carolina Ibáñez, Francisco Acevedo, Jorge Madrid, José Peña, Erica Koch, Maria José Maturana, Diego Romero, Nathaly de la Jara, Javiera Torres, Manuel Espinoza, Carlos Balmaceda, Yuwei Liao, Zhiguang Li, Matías Freire, Valentina Gárate-Calderón, Javier Caceres, Gonzalo Sepúlveda-Hermosilla, Rodrigo Lizana, Liliana Ramos, Rocío Artigas, Enrique Norero, Fernando Crovari, Ricardo Armisén, Alejandro H. Corvalán, Gareth I. Owen and Marcelo Garrido

Table S1. Demographic and clinico-pathological characteristics of HER2+ subgroup (*n* = 12), EBV+ subgroup (*n* = 12), PDL1+ subgroup (*n* = 26) and MSI+ subgroup (*n* = 13) populations.

Characteristic	HER2+ *n* (%)	EBV+ *n* (%)	PDL1+ *n* (%)	MSI+ *n* (%)
n	12	12	26	13
**Gender**				
Male	7 (58.3)	11 (91.7)	21 (80.8)	7 (53.8)
Female	5 (41.7)	1 (8.3)	5 (19.2)	6 (46.2)
**Stage at diagnosis**				
I	2 (16.7)	1 (8.3)	3 (11.5)	2 (15.4)
II	3 (25.0)	5 (41.7)	9 (34.6)	6 (46.2)
III	6 (50.0)	4 (33.3)	12 (46.2)	4 (30.8)
IV	1 (8.3)	2 (16.7)	2 (7.7)	1 (7.7)
**Location of primary tumor**				
Distal esophagus and GEJ	3 (25.0)	6 (50.0)	7 (26.9)	1 (7.7)
Fundus	0	3 (25.0)	3 (11.5)	0
Corpus	4 (33.3)	2 (16.7)	8 (30.8)	3 (23.0)
Antrum	1 (8.3)	1 (8.3)	7 (26.9)	7 (53.8)
Pylorus	1 (8.3)	0	0	1 (7.7)
Multiple	2 (16.7)	0	1 (3.9)	1 (7.7)
NA	1 (8.3)	0	0	0
**Lauren histological type**				
Intestinal	8 (66.7)	4 (33.3)	6 (23.1)	3 (23.0)
Diffuse	2 (16.7)	2 (16.7)	9 (34.6)	6 (46.2)
Mixed	1 (8.3)	1 (8.3)	3 (11.5)	2 (15.4)
NA	1 (8.3)	5 (41.7)	8 (30.8)	2 (15.4)
**WHO histological type**				
Adenocarcinoma	10 (83.3)	9 (75.0)	21 (80.8)	12 (92.3)
Undifferentiated carcinoma	0	2 (16.7)	1 (3.9)	1 (7.7)
Adenosquamous cell carcinoma	0	0	1 (3.9)	0
NA	2 (16.7)	1 (8.3)	3 (11.5)	0
**Signet-ring cell presence**				
No	6 (50.0)	11 (91.7)	21 (80.8)	6 (46.2)
Yes	5 (41.7)	0	4 (15.4)	5 (38.5)
NA	1 (8.3)	1 (8.3)	1 (3.9)	2 (15.4)
**Median/average age at diagnosis (years)**	62.5/60.3 (39–83)	57.0/57.7 (36–75)	59.0/61.3 (33–84)	66.0/65.0 (26–82)
**Median overall survival (months)**	30	NR	66	NR

GEJ Gastroesophagic junction, *NA* Not available, *NR* Not reached.

Table S2. Driver mutations affecting FORCE1 patients.

**Gene**	**Driver Mutation** [a]	**Affected Patients (*n*)**
BRAF	p.D594G	3
CTNNB1	p.D32Y	1
FBXW7	p.R465H	1
KRAS	p.G13D	1
NRAS	p.G12D	2
PIK3CA	p.E545K	2
RHOA	p.Y42C	3
TP53	p.R175H	2
	p.R181C	1
	p.R248Q	2
	p.R248W	2
	p.R273C	4
	p.R273H	1
	p.R282W	1
	p.C135Y	1
	p.C176F	1
	p.Q144H	1
	p.P152L	1
	p.Y205S	1
	p.Y220H	2
	p.V173L	1
	p.V272M	1

[a] Driver mutations identified using sequence-based and structure-based approaches.

Table S3. Actionable mutations affecting FORCE1 patients.

Gene	Druggable Mutation	Drug Associate	Level of Evidence [a]	Cancer Type	Affected Patients (*N*)
ALK	Fusion	Crizotinib, Alectinib, Ceritinib	1	NSC Lung	4
ATM	p.S2017fs	Olaparib	4	All solid tumors	1
	p.L581fs				1
	p.D2725fs				1
BRAF	pD594G	PLX8394	4	All tumors	3
	Fusion	Cobimetinib, Trametinib	3A	Melanoma	1
BRCA1	p.Q1111fs	Rucaparib, Niraparib	1	Ovarian	1
BRCA2	p.E2258*	Talazoparib, Olaparib	2A	Breast/Ovarian	1
CDKN2A	Deletion (CNV)	Abemaciclib, Palbociclib, Ribociclib	4	All solid tumors	2
	p.R131C				1
	p.R87Q				1
	p.Q50*				1
ERBB2	Amplification (CNV)	Trastuzumab	1	Esophagogastric	3
	p.R678Q	Neratinib	3A	Breast	2
FGFR2	p.P253R	AZD4547, BGJ398, Erdafitinib, Debio1347	4	All solid tumors	1
HRAS	p.G12D	Tipifarnib	3A	Head and Neck	1
KRAS	p.Q61H	Panitumumab, Cetuximab	R1	Colorectal	1
	p.G12A	Cobimetinib, Binimetinib, Trametinib	4	All tumors	1
	p.G12D				3
	p.G13D				1
NRAS	p.G12D	Panitumumab, Cetuximab	R1	Colorectal	1
	p.G12D	Binimetinib, Binimetinib + Ribociclib	3A	Melanoma	1
PIK3CA	p.E542K	Buparlisib, Alpelisib, Fulvestrant, Serabelisib, Copanlisib, GDC-0077, Taselisib	3A	Breast	4
	p.K111E				1
	p.G118D				1
	p.T1025A				2
	p.H1047R				1
	p.E545G				3
	p.R88Q				2
PTEN	p.T319fs	GSK2636771, AZD8186	4	All tumors	1
	p.T167fs				1
	p.R335*				1
	p.R173H				1
ROS1	Fusion	Crizotinib	1	NSC Lung	1

[a] Downloaded from OncoKB database *NSC* Non-Small cell, *CNV* Copy number variation.

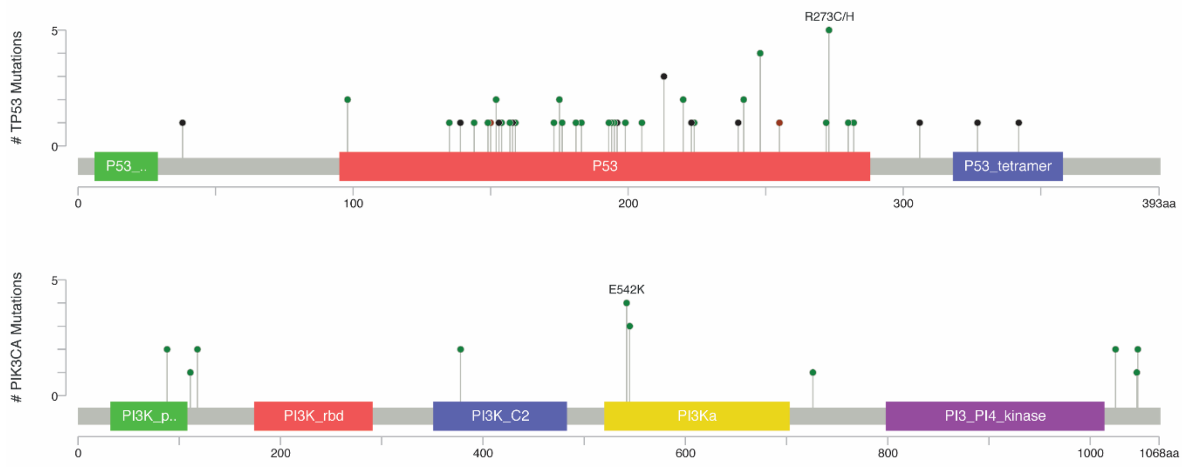

Figure S1. Lollipop diagram showing *TP53* and *PIK3CA* gene mutations found in the GCTF.

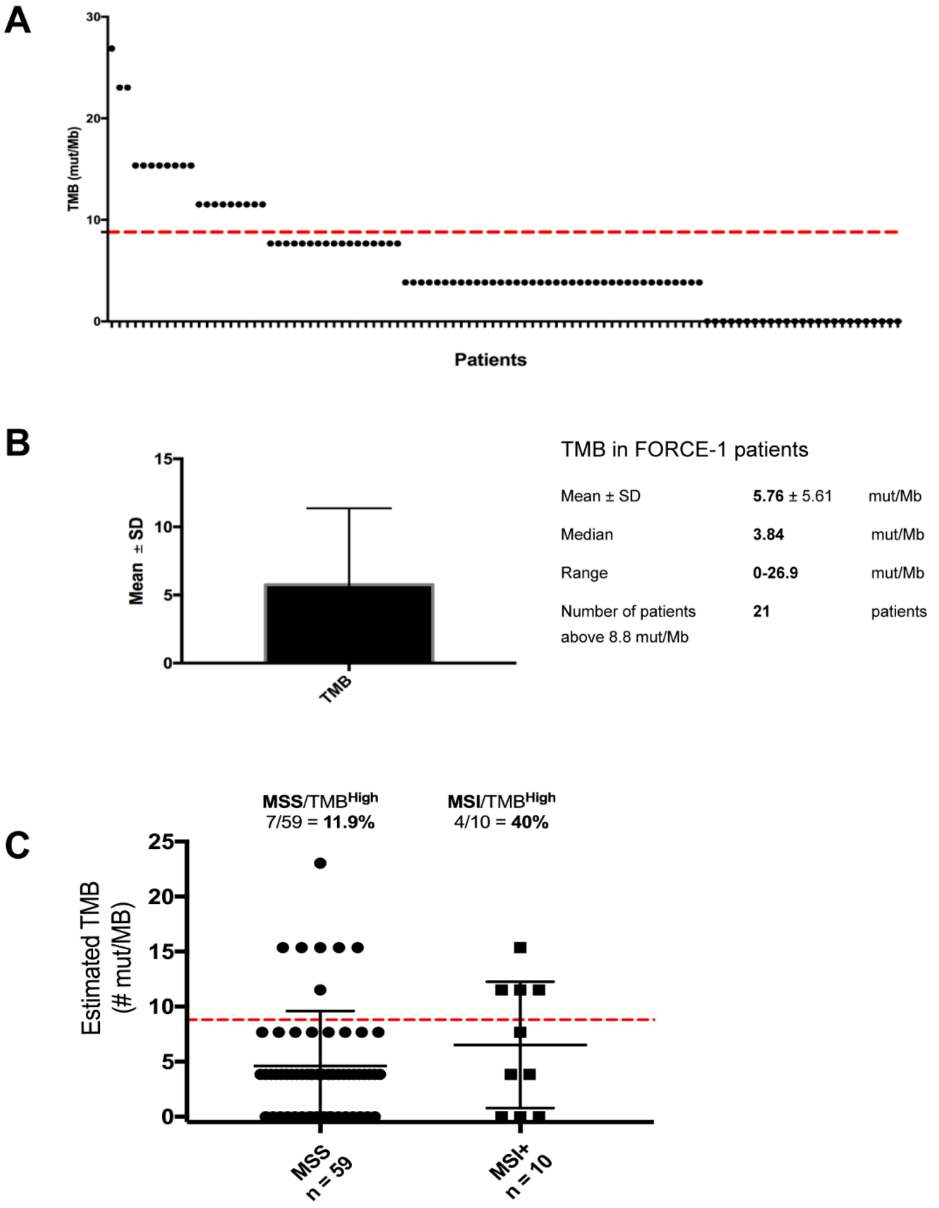

Figure S2. Estimated TMB and TMB levels in MSI+ or MSS patients.

© 2019 by the authors. Licensee MDPI, Basel, Switzerland. This article is an open access article distributed under the terms and conditions of the Creative Commons Attribution (CC BY) license (http://creativecommons.org/licenses/by/4.0/).
